# Prevalence of classes 1 and 2 integrons in multidrug-resistant *Acinetobacter baumanni* isolates recovered from some aquatic environment in South Africa

**DOI:** 10.1038/s41598-022-24724-2

**Published:** 2022-11-24

**Authors:** Oluwaseun Ola Adeniji, Elsiddig A. E. Elsheikh, Anthony Ifeanyin Okoh

**Affiliations:** 1 SAMRC Microbial Water Quality Monitoring Centre, University of Fort Hare, Alice, 5700 South Africa; 2 Department of Applied Biology, College of Sciences, University of Sharjah, P.O. Box 27272, Sharjah, United Arab Emirates; 3 Department of Environmental Health Sciences, College of Health Sciences, University of Sharjah, Sharjah, United Arab Emirates

**Keywords:** Computational biology and bioinformatics, Genetics, Microbiology, Molecular biology

## Abstract

The emergence of antibiotic-resistance genes (ARGs) by means of integrons in multidrug-resistant *Acinetobacter baumannii* (MDR *A. baumannii*) has become a significant challenge in the management of infections from this pathogen. In this paper, we report on the variable region of class 1 and 2 integrons observed in MDR *A. baumanni* isolates recovered from rivers in the Eastern Cape Province, South Africa. Class 1 and 2 integrons with their variable regions were evaluated with polymerase chain reaction techniques followed by sequencing. Antibiotic sensitivity testing, checkerboard assay, time-kill independent assay, and Enterobacterial Repetitive Intergenic Consensus Polymerase Chain Reaction (ERIC-PCR) were carried out using standard microbiological techniques. A total of fifty-six (56) isolates were examined, among which 45 (79%) tested positive for class 1 integron, and 7 (12.3%) had class 2 integron. None was found to be class 3 integron positive among the isolates. The variable region contained *aadA1, aadA5*, and *aadA2* genes, which confer resistance against streptomycin and spectinomycin, *aac(6′)-Ib* against amikacin/ tobramycin and *dfrA17* genes against trimethoprim. The minimum inhibitory concentrations of the antimicrobials for one of the tested organisms were resistant against meropenem, colistin sodium methanesulfonate, tetracycline, ceftazidime, and ciprofloxacin (16, > 16, > 8, > 256, and 128 ug/mL respectively). The impact of colistin combined with quinolones (ciprofloxacin), with the FICIs (0.31) indicated synergistic effects against MDR *A baumanni*. However, when colistin was combined with meropenem and ceftazidime, additive effects with fractional inhibitory concentration (FIC) index ranging from 0.52 to 1 were observed. No antagonistic effect was evaluated among the examined isolates. ERIC-PCR analyses of *A. baumanni* isolates revealed significant genetic diversity, suggesting various sources of environmental contamination. We conclude that *A. baumanni* harbouring class 1 integrons in aquatic milieus are a significant source of ARGs and can transmit these elements to other organisms and consequently to man with significant public health implications.

## Introduction

*A. baumannii* is an important aerobic Gram-negative opportunistic hospital-acquired infections (HAIs) pathogen with wide dissemination in the environment^1^. It is responsible for the diversity of HAIs such as urinary tract infections, ventilator associated pneumonia, bacteraemia, secondary meningitis, surgical-site infections^2,3^, burn wound and soft tissue specifically in the intensive care unit (ICU), and burn units^4^. *A. baumanni*i was listed with the six highest priority pathogenic drug-resistant organisms by the Infectious Diseases Society of America^5^, as its occurrence is becoming a cause for concern globally^6^. Due to the acquisition of a large spectrum of ARGs coupled with environmental adaptation in various harsh conditions by *A. baumannii,* the pathogen is becoming a clinical serious concern^7^. Over the past years, despite novel therapeutic alternatives, *A. baumannii* strains have demonstrated a huge capacity for the speedy growth of multidrug resistance. The increase is not only a result of the intrinsic resistant genes borne by these species but also of their remarkable ability to obtain resistant elements from other microbes^8^. MDR is defined as “resistance to 3 or more unique antimicrobial drug classes, while Extensive drug-resistant (XDR) refers to resistance in all but 1 or 2 antibiotic classes^9^, while Pandrug resistant (PDR) was defined as nonsusceptibility to all agents in all antimicrobial categories”^10^. The primary mode of antibiotic resistance involves enzyme modification, external membrane permeability, alteration in target genes, and an increase in the efflux pump expression^11^.

A novel device for the distribution of antimicrobial resistance genes (ARGs) among microorganisms was described in recent years^12,13^. This mechanism builds on mobile elements such as plasmids transposons and integrons facilitated by horizontal gene transfer^14^. Integrons are viewed as distinctive among these mechanisms for their ability to cluster and express ARGs. The ARGs enhance the capability of *A. baumannii* to be that phenomenally successful disease-causing organism^15,16^. Integrons is a mobile DNA element that can capture several genes by a site-specific recombination mechanism, which frequently carries the gene cassettes containing the ARGs^17^. The core structure of integrons “consists of 5′ and 3′-conserved segments possessing gene cassettes that contain ARGs that could be inserted or excised by a site-specific recombination mechanism catalyzed by the integrase”^8^.

Based on the nucleotide sequence of the integrase gene, six classes of integrons have been detected, under which classes 1, 2, and 3 revealed the main role in transmitting ARGs^18^. Class 1 and 2 integrons are commonly expressed in *A. baumanni*i, play a major role in AR, and usually encode genes for Metallo-β-lactamases resistance, aminoglycoside, β-lactamases, oxacillinases, chloramphenicol, streptomycin, and trimethoprim^19^. The cross-transfer of this bacteria from one patient to another and the likelihood of outbreak extension through patient transmission have been established^20^. In spite of the clinical ecology being arduously studied, its ecology outside the hospital continues to be vague impeding efficient prevention of transmission^21^. Various researchers have the suspicion that the continued existence of *A. baumanni*i in the milieu, particularly in water, could facilitate the transfer of the bacteria during epidemics^22^. In South Africa, *A. baumannii* has been recovered from hospital milieus; however, only a few findings have documented the existence of classes of integrons in *A. baumannii* from the water milieu, which can be a source of their transfer to the hospital environments or may horizontally transfer RGs to other bacteria. Hence, this research evaluates class 1, 2, and 3 integrons with their internal variable regions and combination therapy of MDR *A. baumanni*i isolates from environmental sources in Eastern Cape Province, South Africa.

## Methodology

### Description and geographical map of the sampling sites

**Figure 1:**
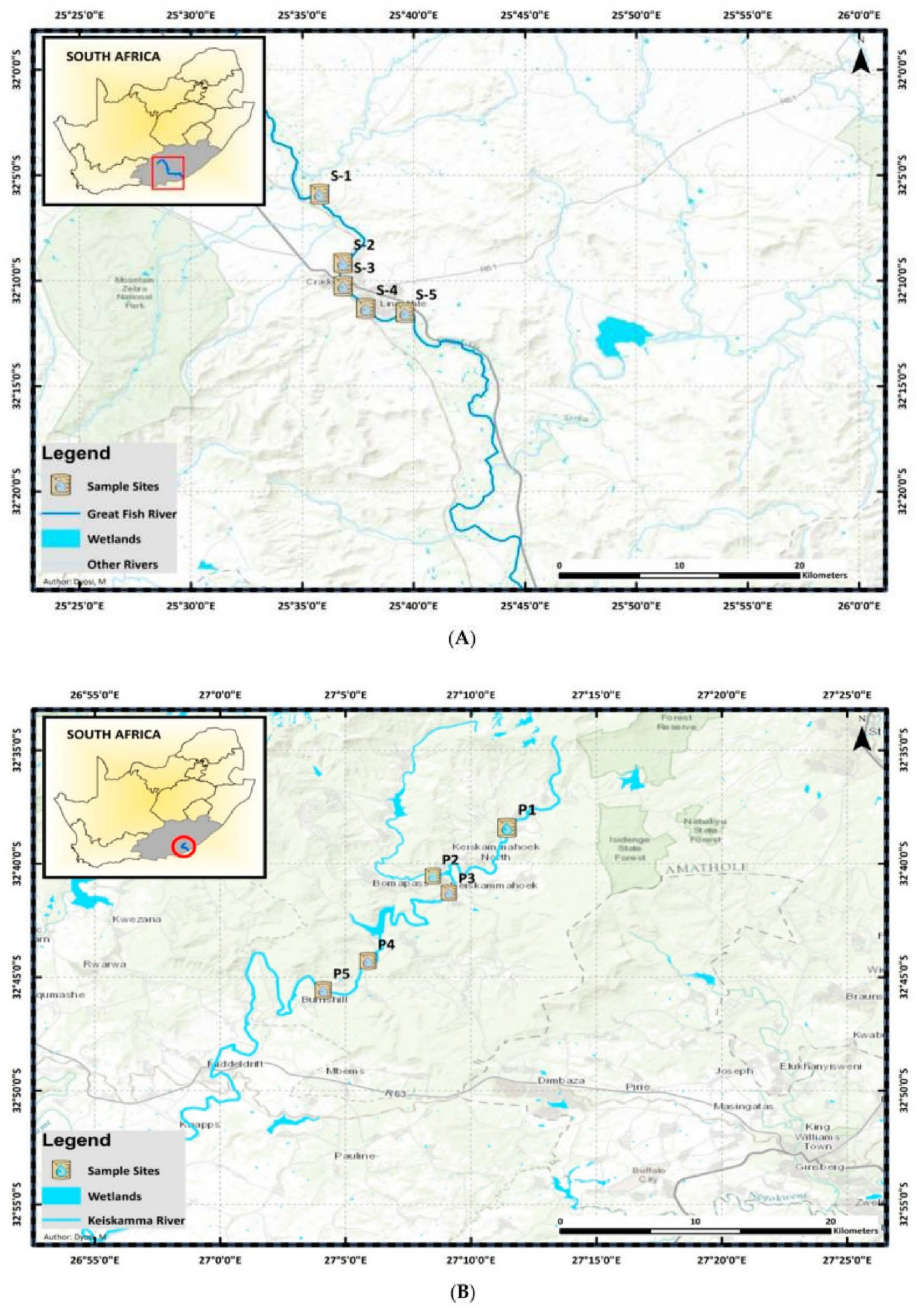

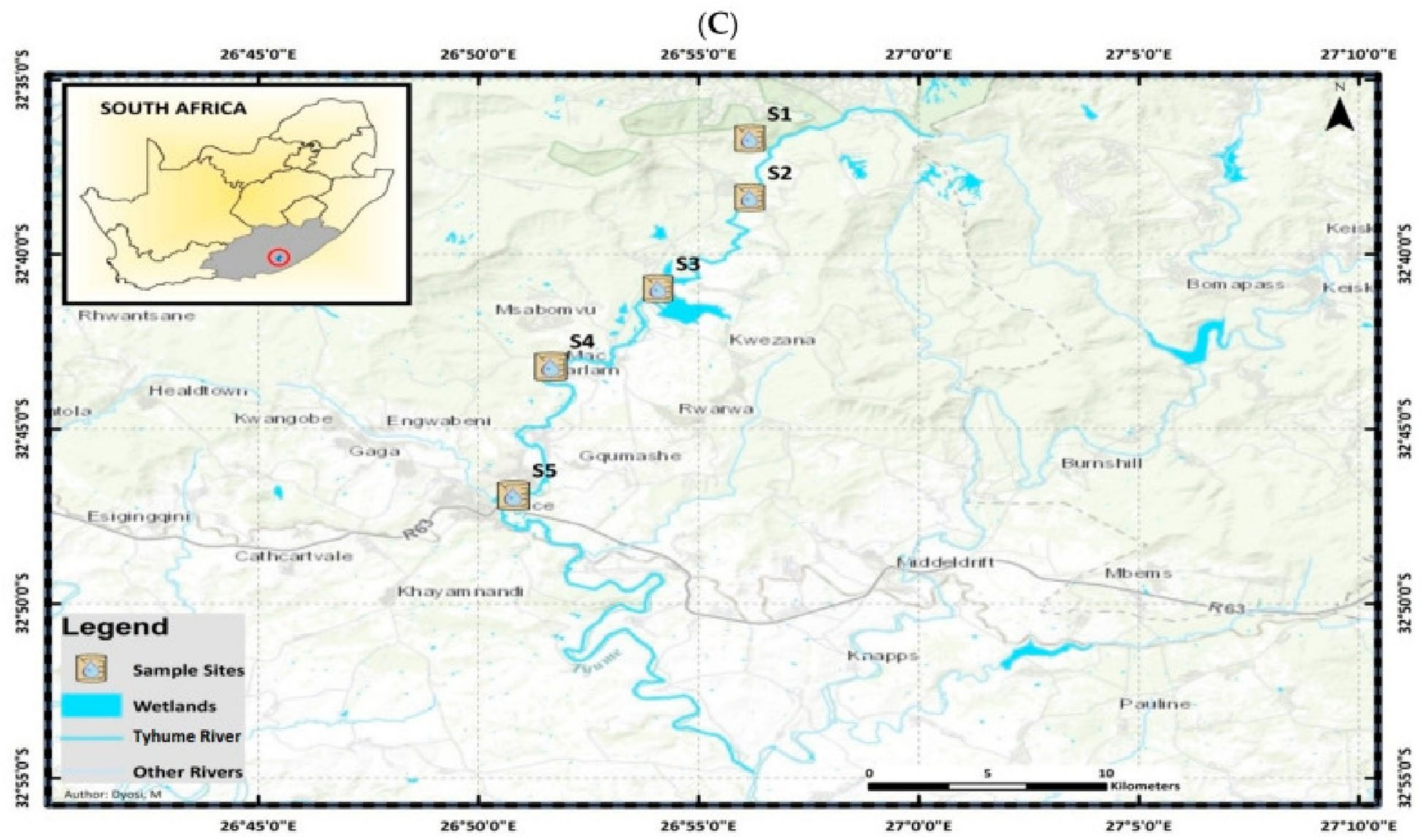
(**A**) Map of Great Fish River showing the sampling points. (**B**) Map of Keiskamma River showing the sampling points (**C**) Map of Tyhume River showing the sampling points [The map has been published in our previous paper by Adewoyin et al. (2021) https://doi.org/10.3390/pathogens10091110 with permission to reuse the maps under a CC BY open access license].

Water samples were collected from three rivers: the Great Fish, Keiskamma, and Tyhume Rivers in the Chris Hani and Amathole District Municipalities of South Africa's Eastern Cape Province. The study chose five sampling points along each river course based on various visible activities in the communities, as shown in Table 1. The coordinates of these rivers and the five sampling points are shown in Fig. 1A–C.

**Table 1:** Anthropogenic activities of the sampling sites.

Site name	Code	Anthropogenic activities
Great Fish River	GF1	Irrigation, fishing
	GF2	Fishing, crop farming, and animal rearing
	GF3	Swimming, animal rearing, and domestic purposes
	GF4	Downstream of the Craddock wastewater treatment plant (WWTP)
	GF5	Fishing, recreational activities
Keiskamma River	KE1	Animal rearing
	KE2	Domestic activities
	KE3	Receives community runoff, wastewater pipe leakage, and domestic refuse
	KE4 & KE5	Downstream of Sandile Dam and a WWTP
Tyhume River	TY1	A recreational site where tourists visit and swim
	TY2	Domestic activities, animal rearing and other farming activities
	TY3 & TY4	Fishing, recreational activities and farming
	TY5	Downstream of hospital waste discharge and waste discharge from the University of Fort Hare

### Bacterial isolation, revitalization, and extraction of genomic DNA

Water samples were collected from the Great Fish, Keiskamma, and Tyhume Rivers in South Africa's Eastern Cape Province (Fig. 1A–C). The presumptive Acinetobacter species was isolated using a selective medium (CHROMagar Acinetobacter base) containing selective supplements (CHROMagar, Paris, France) at 37 °C for 24 h, as recommended by the American Public Health Association^23^. The manufacturer's instructions were followed when preparing the selective medium. Following incubation, distinct *A. baumannii* colonies were subcultured on nutrient agar (Oxoid, Hampshire, UK) using a streak plate method and incubated at 37 °C for 24 h. Fifty percent (50%) glycerol stocks of the pure culture were prepared and stored at − 80 °C. A total of 57 isolates was collected from the Applied and Environmental Microbiology Research Group (AEMREG) culture collection in glycerol stock. The isolates were revitalized on Luria Bethani broth and incubated for 16–18 h at 37 °C, and extraction of genomic DNA was performed by boiling method according to Garrido-Maestu et al.^24^.

### Detection and confirmation of *Acinetobacter baumanni* using PCR

The *gyr*B gene-specific primers of *A. baumanni*i at 208 bp fragment was performed using PCR techniques as described by Chen et al.^25^, with both the forward and reverse primers P-rA1 (5′-CCTGAATCTTCTGGTAAAAC-3′) and P-rA2 (5′-GTTTCTGGGCTGCCAAACATTAC-3′), respectively. The PCR products were verified in 1.5% agarose gel stained with ethidium bromide at 100 V for 40 min and the images were visualized with the aid of an Ultraviolet (UV) transilluminator (Alliance 4.7, France). ATCC 19,606 was used as positive control.

### Estimating genetic variation of *Acinetobacter baumanni* isolates using Enterobacterial Repetitive Intergenic Consensus Sequence PCR

ERIC-PCR was carried out using the DNA extracts and cycling conditions reaction mixture as described by Ateba & Mbewe^26^. The set of primers ERIC1-F:ATGAAGCTCCTGGGGATTCAC and ERIC2-: AAGTAAGTGACTGGGGTGAGCG were used^26^. Amplification products were evaluated with electrophoresis and reviewed on 3% agarose gel stained by ethidium bromide. Visualization of the gel was done as described in Sect. "Detection and confirmation of Acinetobacter baumanni using PCR" above. Digitization of the clonal affinity between *A. baumanni* was achieved via a GelJ version 2.0.

### Antibiotic sensitivity Testing

Antibiotic sensitivity testing was done using the Kirby–Bauer disk diffusion protocol as described in the CLSI procedures for the following antibiotics^27^: Amikacin (30 µg), Cefotaxime (30 µg), Cefepime (30 µg), Ceftazidime (30 µg), Gentamicin (10 µg), Ciprofloxacin (5 µg), Imipenem (10 µg), Meropenem (10 µg), Tetracycline (30 µg), Piperacillin tazobactam (100 µg), and sulphamethoxazole (MAST, Merseyside, UK). Isolates that are non-susceptible to three-or-more antibacterial drug groupings are classified as MDR while those that are resistance in all but 1 or 2 antibiotic classes are classified as Extensive drug-resistant (XDR) *A. baumannii*^9^. *A. baumannii* “ATCC 19606” was used as a positive control.

### Amplification of class 1, 2, and 3 and internal variable region genes

The presence of integrase gene *intI1*, *intI2*, *intI3* in MDR *A. baumanni* was investigated in each one of the isolates by means of PCR with specific primers (Table 2). Afterward, all integron positive MDR *A. baumanni* strains were screened for the occurrence of internal cassettes genes using 3′CS and 5′CS primers as shown in Table 2. All primers were obtained from Inqaba Biotech (South Africa). The preparation of PCR reactions and amplification was carried out as mentioned by^31–34^, respectively. PCR product was amplified and visualized as described previously.

**Table 2:** List of primers used in this study.

Target gene	Primer sequence (5′-3′)	Amplicon size (bp)	Reference
*IntI1*	F: “CAG TGG ACA TAA GCC TGT TC” R: CCC GAG GCA TAG ACT GTA	I64	^28^
*IntI2*	F: “TTATTGCTGGGATTAGGC” R: “ACGGCTACCCTCTGTTATC”	232	^29^
*IntI3,*	F: “AGTGGGTGGCGAATGAGTG” R: “TGTTCTTGTATCGGCAGGTG”	600	^30^
*5′CS* *3′CS*	“5′CS-F GGCATCCAAGCAGCAAG” “3′CS-R AAGCAGACTTGACCTGA”	Variable	^31^
*attI2* *orfX*	“attI2- “GACGGCATGCACGATTTGTA” “orfX “RGATGCCATCGCAAGTACGAG”	Variable	^31^

### Integrons gene cassettes analysis and DNA sequencing

The sequencing of the purified PCR fragments was done with “the Nimagen, BrilliantDye TERMINATOR CYCLE Sequencing Kit V3.1, BRD3-100/1000” as clearly stipulated in the manufacturer's instructions.

### Phylogenetic analysis

The “nucleotide sequences of 16S rRNA genes, and the amino acid sequences for *A. baumanni* were aligned using the ClustalW2 alignment instrument. Evolutionary trees were constructed using the neighbor-joining (NJ) method with the Kimura-2-parameter model, maximum likelihood (ML) with the JTT model, and maximum parsimony (MP) in the MEGA7 package program^32^. Phylogenetic confidence was evaluated by the non-parametric bootstrap method with 1000 replicates”^33^.

### Experimental drugs and main instruments

The drugs used in these tests were bought from Sigma-Aldrich and the details about the preparation of antibiotics are presented in Table 3.

**Table 3:** The list of antibiotics used for MIC with their solvent and diluent (Andrews^34^).

Antimicrobial class	Antibiotics	solvent	Diluent
Cephalosporins	Ceftazidime hydrate	0.1 M saturated NaHCO_3_ solution	Water
Polymyxins	Colistin sodium methanesulfonate	Water	Water
Quinolones	Ciprofloxacin	Water	Water
Carbapenems	Meropenem	Water	Water
Tetracyclines	Tetracycline	Water	Water

### Evaluation of minimal inhibitory concentration (MIC)

The minimum inhibitory concentrations (MICs) for the antibiotics under study were determined in triplicate using round-bottomed 96-well microtiter plates (Greiner Bio-one, Monroe, NC, USA) and the microdilution method according to CLSI standards^28^. The MICs for ceftazidime were 1 to 512 µg/ml, 0.125 to 64 µg/ml for colistin sodium methanesulfonate, 1 to 2048 µg/ml for tetracycline, 0.06 to 128 µg/ml for Meropenem, and 0.5 to 256 µg/ml for ciprofloxacin. Following that, one hundred microliters (100 µL) of the highest concentration of the drug was placed in each well of Column 1. Columns 2–10 contained only diluents, while Column 11 contained 100 µL of diluted standardized inoculum as a growth control and Column 12 contained 100 µL of the Muller Hilton broth as sterility control. Antibiotics from columns 1–10 were mixed and transferred using a micropipette, yielding 50 µL antibiotics per well (serial two-fold dilution). The standardized inoculum suspension was then diluted in MHB by 1:100. (0.1 mL into 9.9 mL of MHB). Fifty microliters (50 µL) of the adjusted OD600 bacterial suspension was added to each well containing different antibiotic concentrations as well as the control wells, yielding approximately 1 × 10^6^ CFU mL. To avoid a change in cell number, the time taken to prepare and dispense the standardized inoculum did not exceed 30 min. After incubating for 24 h at 37 °C, resazurin (0.015%) was added to all wells (30 L per well) and incubated for another 2–4 h to observe color change. The MIC was recorded as the concentration of antimicrobial agent that inhibits visible color change in broth medium wells. *Staphylococcus aureus* ATCC 29213 and *Pseudomonas aeruginosa* ATCC 27853 were used as reference organisms to validate the performance of each antibiotic stock solution. The reference strains were procured from the American Type Culture Collection (USA). The results were compared with EUCAST values^35^

### Synergistic antimicrobial assays

Evaluation of fractional inhibitory concentration index (FICI) was carried out using checkerboard assay as documented by Petersen et al.^36^. The MDR *A. baumannii* strains were verified against combinations of two drugs using bacterial inoculum of 5 × 10^5^ CFU/mL, the test was done twice for each (duplicate) and examined after 24 h of incubation at 35 °C. Sterility and growth controls were tested on all plates. Colistin and ceftazidime were combined with ciprofloxacin, tetracycline, and meropenem at the respective MIC determined previously. These antibiotics were selected to represent each major antibiotic class and each has a different mechanism of action including inhibition of targets in different pathways, inhibition of targets in the same pathway, and inhibition of the same target in various ways. FICIs was calculated as the “[(MIC of drug A in combination)/(MIC of drug A alone)] + [(MIC of drug B in combination)/(MIC of drug B alone)]. Synergy was defined as a FICI of ≤ 0.5, indifference as a FICI of > 0.5 < 4, and antagonism as a FICI of ≥ 4”^37^.

#### Time kill assay

Time-kill analyses were conducted solely on drug combinations found to show a synergistic effect with the checkerboard assay as previously published in the CLSI^28^. Briefly, colistin sodium methanesulfonate concentrations ranged from 0.125 to 64 g/ml, and ciprofloxacin concentrations ranged from 0.5 to 256 g/ml. A synergistic effect was observed when colistin at 32 g/ml and ciprofloxacin at 128 g/ml MICs were combined. Each drug was diluted to concentrations of ^1^/_2_MIC, MIC and 2 MIC. The combined antibiotics at 10 mL each were tested in a 100 mL sterile flask. Mueller Hinton broth without test organisms was used as growth control. One hundred microliters of the adjusted 0.5 McFarland inoculum was then mixed with 10 mL of MHB to yield a final concentration of 5 × 105 CFU/mL The cultures were incubated at 35 °C for 18 h with shaking at 120 revolutions per minute (rpm). Aliquots were taken from the cultures at 0, 2, 4, 6, 8, and 24 h. A tenfold dilution series was carried out in sterile MHB after which one hundred microliters of each suitable dilution were applied in triplicate on MHA plates. Parallel to each experiment, a growth control was performed. The mean colony counts (log CFU/mL) were plotted against the incubation times to produce the time-kill curves. When there was a decrease of 3 log10 CFU/mL compared to the original inoculum, the efficacy of the combination therapy was also assessed as bactericidal.

#### Statistical analyses

Bivariate analyses in SPSS version 21 were used to assess correlations between study variables. The heatmaps were created in Microsoft Excel (Microsoft office 2016) to emphasize the value of antibiotic resistance phenotypes and Integron types.

## Results

### Antibiotic sensitivity testing

**Figure 2:**
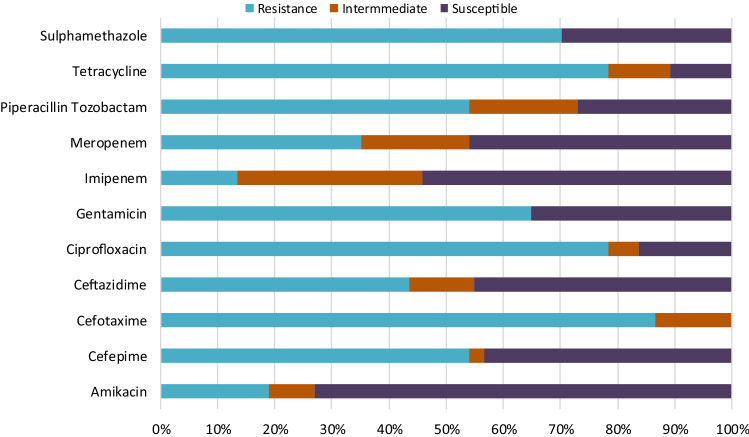
Antibiotic sensitivity pattern of class 1 integron MDR *Acinetobacter baumanni.*

Forty-five **(**45) isolates revitalized for identification through polymerase chain reaction were all positive for *Acinetobacter baumanni*; Gel electrophoresis image is shown in Fig S1. About 37/45 (82%) *Acinetobacter baumanni* screened for antibiotic sensitivity were MDR organisms resistant to at least one agent in three or more antimicrobial classes^9^. The MDR isolates showed 72.97%, 45.9%, 54.1% susceptible phenotype in response to Amikacin, meropenem and imipenem, respectively. The percentage of isolates resistant against tested antibiotics included gentamicin (64.9%), ceftazidime (51.4.7%), cefotaxime (86.5%), cefepime (54.1%), tetracycline (78.4%) ciprofloxacin (78.4%), piperacillin tozobactam (54.1%) and trimethoprim/sulfamethoxazole (70.3%) as shown in Fig. 2.

### Integron appraisal of *A. baumannii* strains

**Figure 3:**
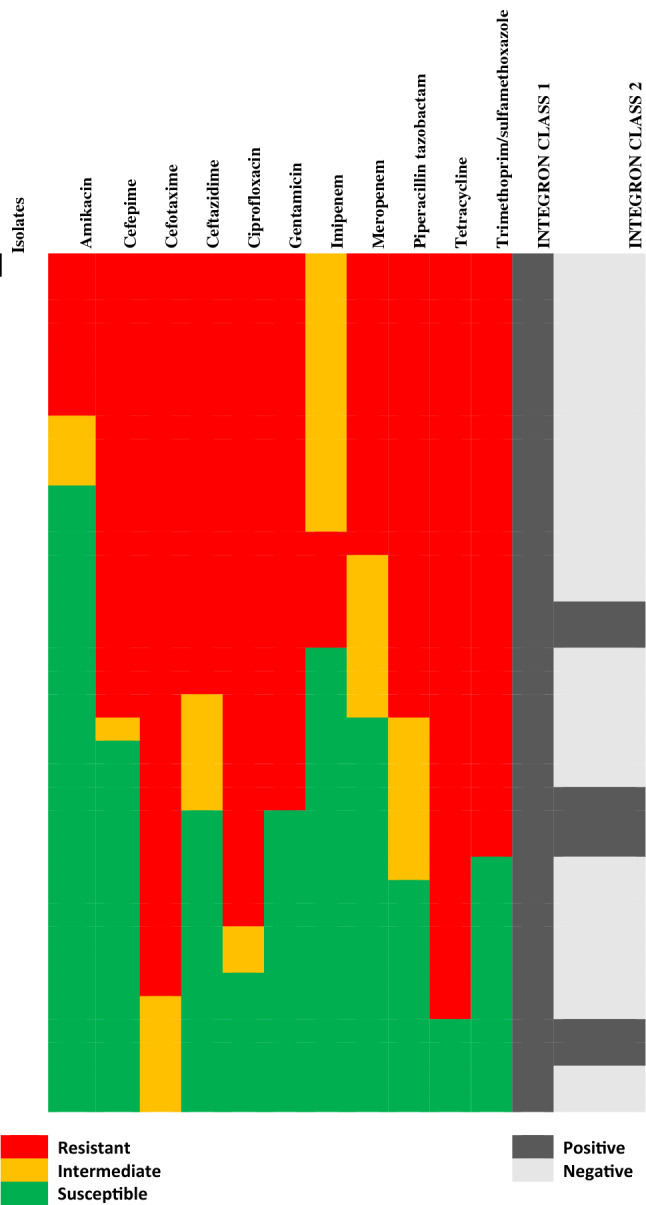
The heat map representing correlations among antibiotic resistance profile in *Acinetobacter baumanni* and integron type.

Of the integron-positive isolates, 45 (79%) were evaluated to be class 1, while 7 (12.3%) were class 2 integrons. We did not evaluate class 3 integron in the environmental samples. The gel image is shown in Figs. S2 & S3 in the supplementary. The heat map of multidrug integron habouring *A. baumanni* is shown in Fig. 3 below.

### Mapping of integrons

**Figure 4:**
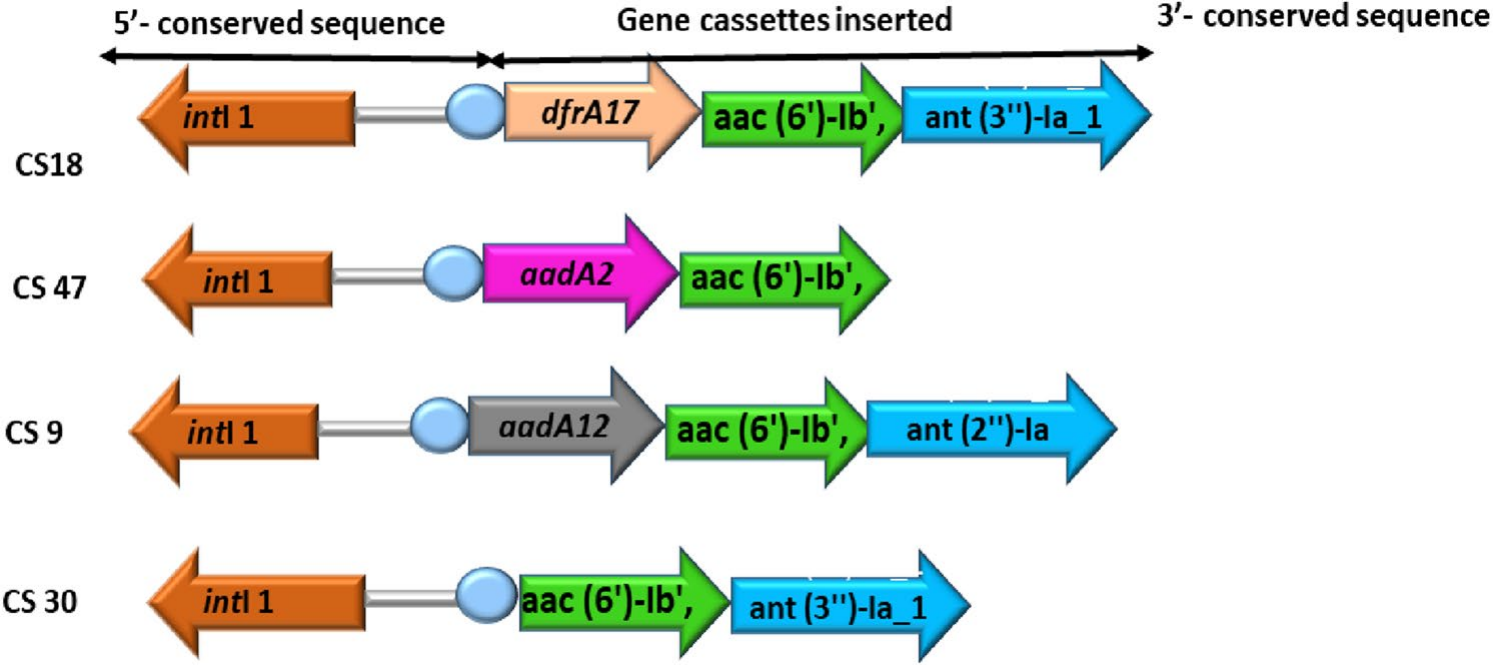
Schematic illustration of the gene cassette arrays within class 1 evaluated from *A. baumanni* from this study. “(dfrA12), dihydrofolate reductase; aminoglycoside 6'-N-acetyltransferase gene (*aac (6')-Ib*′) genes, Aminoglycoside-2″-O-nucleotidyltransferase (*ant (2'')-Ia* and aminoglycoside (3'') (9) adenylyltransferase (aadA2) genes”.

The variable region of integrons was discovered in four *Acinetobacter baumanni* isolates amongst the 46 class I integron-positive isolates, which have been characterized by sequenced amplification products. Six different types of genes were detected. Obtained DNA sequences were determined using the BLAST program which is accessible on NCBI website (http://www.ncbi.nlm.nih.gov/BLAST/), Resfinder, Card, and Argannot database. Gene cassettes encrypting the dihydrofolate reductase genes (*dfrA*17) that “confer resistance to trimethoprim, and those coding aminoglycoside adenyltransferase genes (*aadA*2, *aac* (6')-Ib', *ant (3'')-Ia_1, aadA*5, *an*t (2'')-Ia and *aadA*12) responsible for resistance to streptomycin/spectinomycin, were also found among the isolates (Table 4). The schematic view of the gene cassette array is presented in Fig. 4.

**Table 4:** Length product and gene cassette results of A. *baumannii* strains.

Bacterial strains	Primer pair	Length of product (bp) Projected Observed	Gene cassette	Accession number
*Acinetobacter baumanni* [CS9]	5’-CS and 3’-CS	1000 907	*aadA2,* *aac (6')-Ib'*	ON622475
*Acinetobacter baumann*i [CS18]	’’	1200 1477	*aac (6')-Ib',* *ant (3'')-Ia_1* *dfrA17, aadA5*	ON622476
*Acinetobacter baumann*i [CS 30]	”	800 755	*ant (2'')-Ia* *aac (6')-Ib'*	ON622477
*Acinetobacter baumanni* [CS 47]	”	900 1260	*aadA12* *aac (6')-Ib* *ant (2'')-Ia*	ON622478

### Minimum inhibitory concentration results for *intI*1-positive Acinetobacter* baumanni*

The MIC ranges of colistin sodium methanesulfate, meropenem, tetracycline, ceftazidime, and ciprofloxacin were > 16, > 16, > 8, > 256, and 128 ug/mL, respectively, as shown in Table 5. All the tested isolates were integron class 1 positive and MDR organisms. They were all resistant to ceftazidime, colistin, tetracycline, and ciprofloxacin. Even though colistin could be the last resort for serious infections in severely ill patients, it demonstrated antibacterial action against MDR gram-negative disease causing organisms. Nevertheless, there is considerable proof that it does not elude resistance.

**Table 5:** Distribution of minimal inhibitory concentration (MIC) of MDR *A. baumannii.*

Pathogens	ceftazidime (µg/ml)	Colistin sodium methanesulfate (µg/ml)	Tetracycline (µg/ml)	Meropenem (µg/ml)	Ciprofloxacin (µg/ml)
MDR *A baumanni* (30)	256	16	8	1	128
MDR *A. baumanni* (44)	512	32	2048	16	128

### Checkerboard assay

The checkerboard assay results of the double combinations for synergistic effects among the selected strains of *A. baumannii* by the broth microdilution checkerboard method are shown in Table 6. The impact of colistin combined with quinolones (ciprofloxacin) with the FICIs (0.31) indicated synergistic effects against MDR *A baumanni* (44). However, when colistin was combined with meropenem and ceftazidime, additive effects with FIC, ranging from 0.52 to 1 on tested strains was observed. No antagonistic effect was evaluated among the examined strains.

**Table 6:** Summary of antibiotic combination therapy results for the studied organisms.

Antibiotic combination	MDR *Acinetobacter baumanni* (30) FIC	Type of interaction	MDR *Acinetobacter baumanni* (44) FIC	Type of interaction
Ciprofloxacin + Colistin	1	Additive	0.31	Synergistic
Ceftazidime + Colistin	1	Additive	0.52	Additive
Ceftazidime + Ciprofloxacin	0.75	Additive	0.52	Additive
Tetracycline + Meropenem	1	Additive	0.75	Additive
Tetracycline + Colistin	0.56	Additive	0.75	Additive
Colistin + Meropenem	1	Additive	0.53	Additive
Ceftazidime + Meropenem	1	Additive	0.75	Additive
Ciprofloxacin + Meropenem	1	Additive	0.53	Additive

### Time-kill study

**Figure 5:**
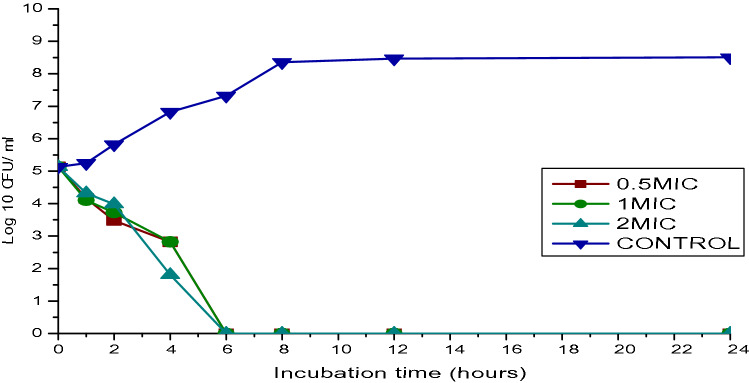
Time kill assay of Ciprofloxacin with Colistin sodium methanesulfonate antibiotics against *Acinetobacter baumanni*.

The combination of colistin at 32 µg/ml and ciprofloxacin at 128 µg/ml of the MIC expressed bactericidal activity at the 4 h at 2 MIC, and 6 h of incubation for 0.5MIC and 1MIC (Fig. 5). However, bactericidal activity occurred faster at 2MIC. The bactericidal effect is the reduction of at least 3 log10 CFU/mL in the viable cell counts concerning the initial inoculum (99.9% killing) and bacteriostatic activity has been defined as a reduction of less than 3 log10 CFU/mL in viable cell counts (90–99% killing.

### ERIC PCR analysis for MDR *Acinetobacter baumanni*

**Figure 6:**
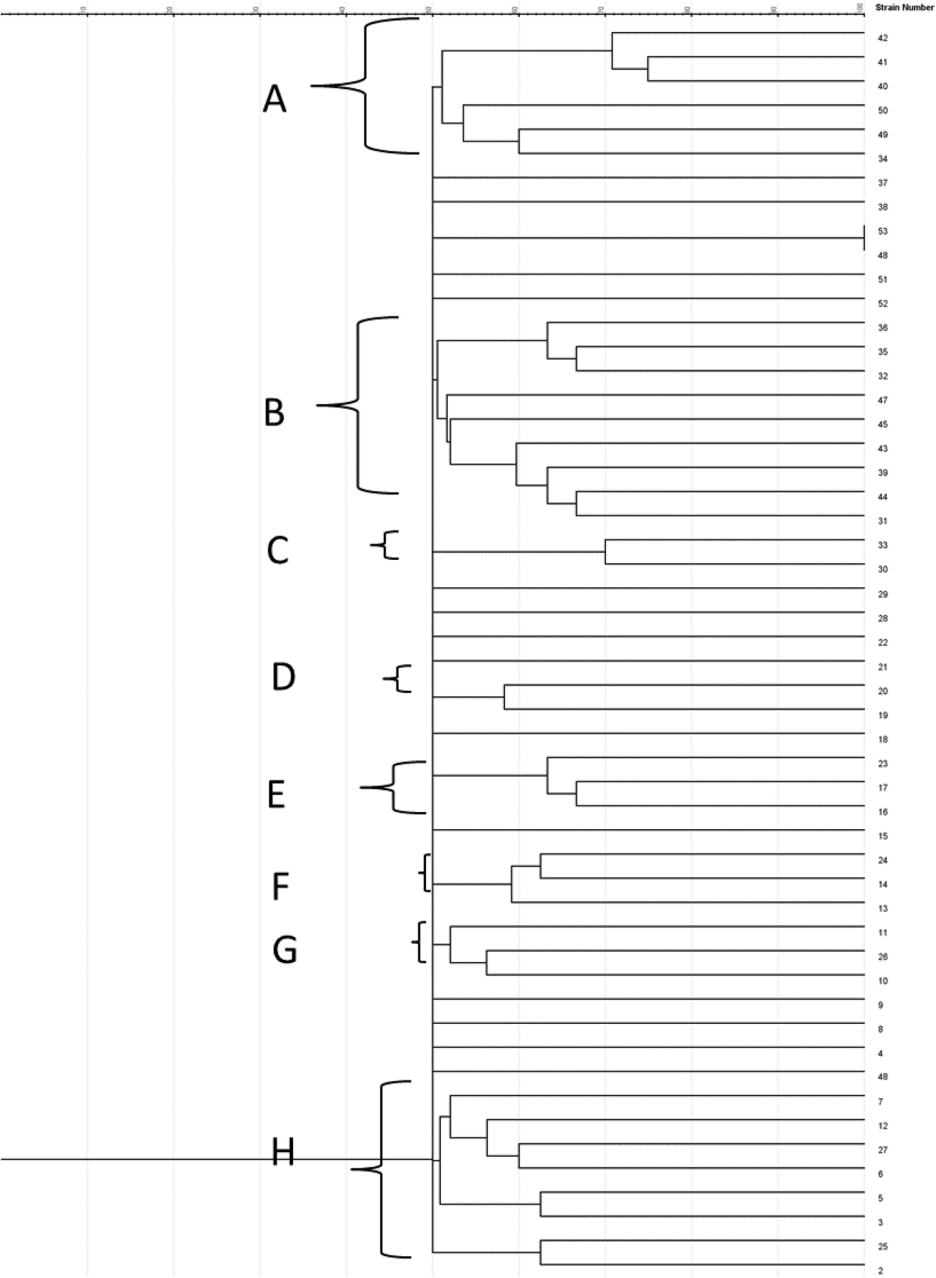
Dendrogram of ERIC PCR analysis for *A. baumanni* from environmental sources.

The outcome of this survey indicates an impact of the epidemiology similarity on the grouping of 36 isolate *A. baumannii* were clustered into eight clades, while the rest eleven were single strains (Fig. 6). Based on our findings, arguably ERIC-PCR is a reliable way to show the clonal affinity among *A. baumannii* of environmental sources. The genetic similarity of *A. baumannii* recovered from the aquatic environment was high suggesting cross transmission within the samples.

### Phylogeny analysis tree for the strains of *Acinetobacter baumanni* based on the amino acid sequences of internal gene cassette

**Figure 7:**
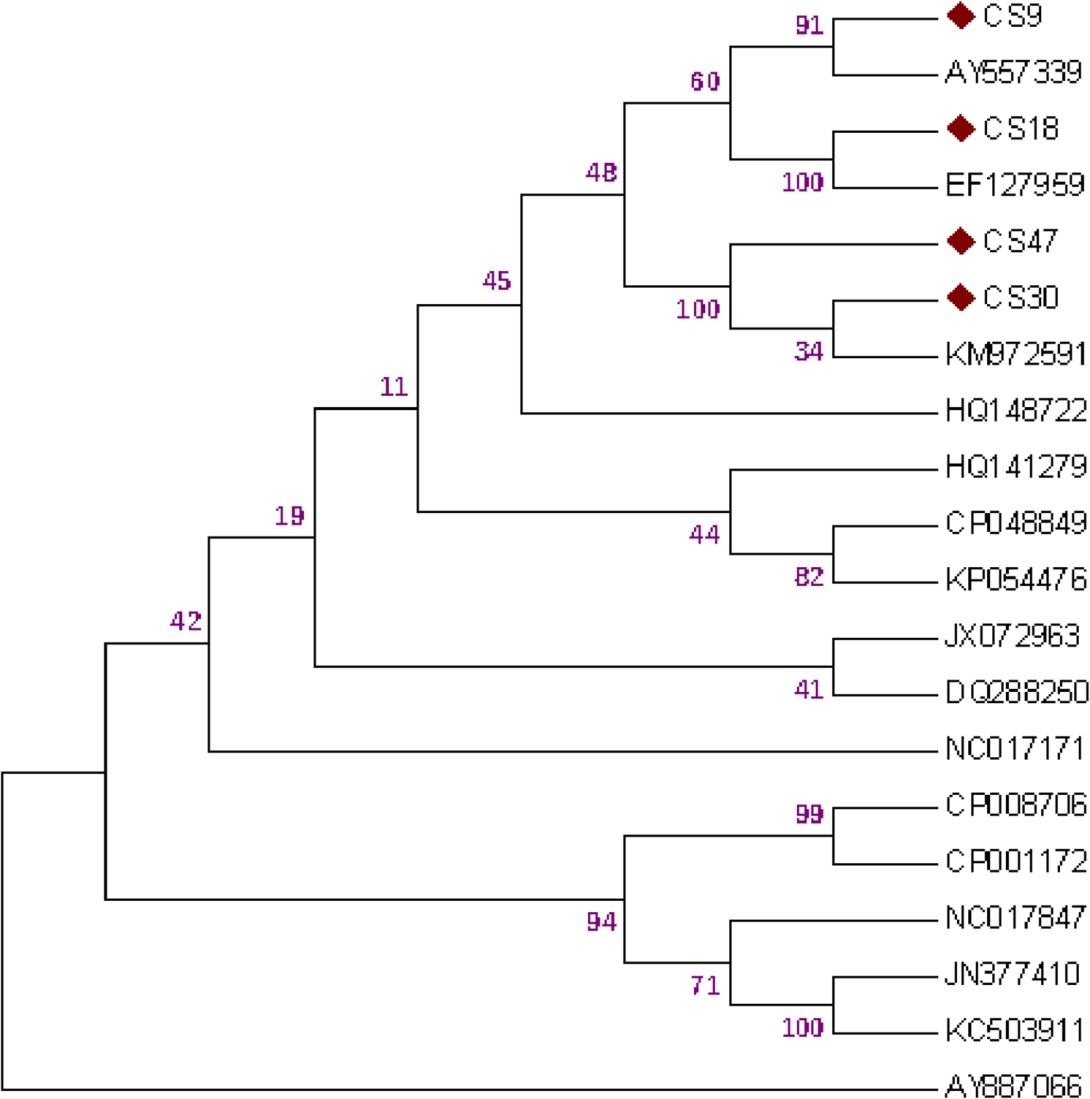
Phylogenetic tree for the strains of Acinetobacter based on the amino acid sequences of internal gene cassette by the neighbor-joining method.

The homologous search of the generated sequences turned out to be high proportion of identity between 98 and 100% with other homologous sequences of other *A. baumanni strains* in GenBank (Fig. 7). Sequences were aligned using ClustalW2, and evolutionary history was derived with the use of “Neighbor-Joining method^38^. The bootstrap consensus tree extrapolated from 1000 replicates^33^ is made to represent the evolutionary history of the taxa assessed^33^ Branches that correspond to partitions reproduced in less than 70% bootstrap replicates are collapsed”. The analysis involved 20 nucleotide sequences. All positions which contained gaps and missing data were removed. Evolutionary assessments were performed on MEGA7^39^.

## Pearson correlation between antibiotics in association with integron 

Pearson correlation analysis revealed a positive and significant correlation (*p* < 0.05) between amikacin and cefepime, ceftazidime, gentamicin, meropenem, and piperacillin tazobactam but moderate correlation with trimethoprim/sulfamethoxazole and no correlation at all with tetracycline, ciprofloxacin, and cefotaxime (Table 7). Except for cefepime and meropenem, which exhibited moderate correlation with tetracycline, and cefotaxime with the duo of imipenem and meropenemthe, the results generally indicate that each antibiotic demonstrates strong positive correlation with one another. Imipenem also did not show any form of correlation with tetracycline. Notably, all the tested antibiotics did not correlate positively with class two integrons, and this could be because the occurrence of class 1 integrons is more common in gram-negative organisms, particularly the pathogen under study. Ghazalibina et al.^40^ explained in their findings that class 1 integrons are more common than other classes of integrons due to their location on genetic elements such as conjugative plasmids and transposons (Table 8).

**Table 7:** Pearson correlation showing a relationship among the antibiotics and class 2 integron in the studied pathogens.

Antibiotics	Amikacin	Cefepime	Cefotaxime	Ceftazidime	Ciprofloxacin	Gentamicin	Imipenem	Meropenem	Piperacillintazobactam	Tetracycline	Trimethoprim/sulfamethoxazole	Integron Class 2
Amikacin	1											
Cefepime	0.528**	1										
Cefotaxime	0.229	0.447**	1									
Ceftazidime	0.531**	0.944**	0.502**	1								
Ciprofloxacin	0.294	0.572**	0.857**	0.642**	1							
Gentamicin	0.427**	0.832**	0.537**	0.934**	0.687**	1						
Imipenem	0.329*	0.756**	0.329*	0.761**	0.421**	0.612**	1					
Meropenem	0.719**	0.908**	0.394*	0.880**	0.505**	0.734**	0.692**	1				
Piperacillin tazobactam	0.493**	0.928**	0.585**	0.941**	0.748**	0.891**	0.706**	0.848**	1		\	
Tetracycline	0.202	0.393*	0.881**	0.442**	0.755**	0.473**	0.289	0.347*	0.515**	1		
Trimethoprim/sulfamethoxazole	0.377*	0.735**	0.608**	0.825**	0.778**	0.884**	0.541**	0.649**	0.893**	0.535**	1	
Integron Class 2	0.280	0.124	0.011	0.161	0.125	0.223	0.016	0.250	0.232	0.054	0.290	1

**Correlation is significant at the 0.01 level (2-tailed).

*Correlation is significant at the 0.05 level (2-tailed).

**Table 8:** Logistic regression analysis for significant predictors of the presence of integron Variables in the Equation.

		B	S.E	Wald	df	Sig	Exp(B)
Step 0	Constant	− 1.455	0.420	12.020	1	0.001	0.233

*B* Regression coefficients, *S.E.* standard error around the coefficient for the constant, *Wald* Wald chi-square test statistic, *Sig.* *P* value for Wald test.

## Discussion

The dissemination of MDR *A. baumannii* strains with high non-sensitivity against various classes of drugs has become a major concern^41^. Immunity to antibiotics is often associated with the horizontal transfer of ARGs through mobile elements^42^. Thus, the advent of the ARGs out of integrons in MDR *A. baumannii* isolates is now a big challenge in treating diseases from these pathogens. Our findings revealed a significant percentage of MDR *A. baumannii* isolates (82%) that were non-susceptible to multiple antibiotics agents used in treating *A. baumannii* diseases. Likewise, in a report by Taitt et al.^43^ in the United States, about 80% of *A. baumannii* isolates were MDR, which agrees with the findings in this study.

Abdar et al.^44^ obtained high resistance of *A. baumanii* to meropenem (71%) and ceftazidime (93%) in their study; however, in this study, a reduction in resistance to both drugs was evaluated. The high rate of resistance to tetracycline and ciprofloxacin could be related to the use of these drugs as growth promoters; hence, they eventually find their way to the aquatic milieu^45^. Gurung et al.^46^ reported that tetracycline represents over 30% of the aggregate amount of antibiotics used often in the treatment of farm animals. Likewise, the high rate of non-susceptibility of cephalosporins may be due to its common usage in the hospital and could end up in the aquatic milieu through sewage disposal.

Goudarzi et al.^47^ revealed that seventy-four percent (74.1%) and twelve percent (12.5%) of *A. baumannii* strains in their study were positive class 1 and 2 integrons, correspondingly. This is in accordance with our findings. In this research work, we evaluated “aminoglycoside adenylyltransferase genes, which bestow resistance to streptomycin and spectinomycin including *aadA12*, *aadA5*, *ant (2'')-Ia*, *aac (6*′*)-Ib*′*, and ant (3'')-Ia_1”* within the integron structures. In addition, we identified *DfrA17* a dihydrofolate reductase facilitating the drug resistance to trimethoprim. This integron cassette has been found in other parts of China, including, Nanjing and Zhejiang^54,55^ The *dfrA17* and *dfrA12* were detected among gram negative organisms that bore class 1 integrons in the USA^48^, this demonstrates that these variants are prevalent amongst cassettes of class 1 integrons worldwide. Previous research described “*aadA2*, *aacA4-cmlA1*, *dfrA17-aadA5* and *dfrA12*-*orfF*-*aadA2*” genes as commonly detected gene cassettes in both the clinical and the ecological strains^49,50^. In studies performed in Taiwan, *aadA* genes variants, which include “*aadA1, aadA2,* and *aadA4”* were reportedly found in most of MDR *A. baumannii* strains^51,52^

Remarkably, a comparable array, *aac (6')-Ib*′*,* was reported in *P. aeruginosa and P. fluorescens* (GenBank accession no. AY660529), *aadA* was also documented in aminoglycoside adenylyltransferase gene coded by plasmids and integrons in *K. pneumoniae, Corynebacterium glutamicus, “Salmonella spp., C. freundii,* and *Aeromonas Spp” (*GenBank accession no. AF156486). In *A. baumannii* isolate CS18 gene cassette array, “*DfrA17* is an integron dihydrofolate reductase” gene detected in *E. coli* (Accession no. DQ838665), while *aadA5* gene coded by plasmids, transposons, and integrons were found in “*E. coli, K. pneumoniae, Kluyvera georgiana, P. aeruginosa and E. cloacae”* (GenBank accession no. AF137361). The *ant (3'')-Ia_1* gene in this study has 99.25% identity to plasmid or integron encrypted “nucleotidylylation of 2-deoxystreptamine aminoglycosides at the hydroxyl group of position 2″ in *K. pneumoniae, P. aeruginosa, Morganella morganii, E. coli,* and *S. typhim ”* (GenBank accession no. AF 078527).

In this study, the minimum inhibitory concentration values of colistin sodium methanesulfate, meropenem, tetracycline, ceftazidime, and ciprofloxacin were > 16, > 16, > 8, > 256, and 128 ug/mL, respectively. All colistin-non-susceptible isolates were resistant to other drugs, including meropenem, cephalosporins, fluoroquinolones, and tetracyclines. Kipnis and Guery^53^ reported colistin as “a polypeptide antibiotic of the group E polymyxin family. It exhibits rapid and concentration-dependent bactericidal activity by destroying the outer membrane of Gram-negative bacteria. In the mechanism of colistin resistance in Gram-negative pathogens, modification of lipid A, a component of LPS, with the addition of 4-amino-4-deoxy-l-arabinose (Ara4N) or/and phosphoethanolamine is considered” as documented by Raetz & Whitfield^54^.

This deficiency of efficient therapeutics has prompted the trial of combinations of existing agents for synergy activities to combat drug-resistant isolates. Combination treatment has been regarded as a good way to fight MDR *A. baumanni*i^55^. The current study combined colistin, a last-line drug for colistin-resistant MDR isolates, with four other drugs that are not effective against *A. baumanni.* None of the drug combinations was antagonistic. They showed synergistic and additive interactions. Consequently, these combinations can be used in clinical practices.

The dendrogram (Fig. 6) derived from this study demonstrated that the isolates were split into eight clades using the ERIC-PCR procedure. The results showed high genetic variation between *A. baumanni* isolates investigated. Clades formed by *A. baumanni* isolates from ecological water samples revealed an evolutionary relatedness amongst the isolates. The proof of genetic variability among *A. baumanni* isolates identified from surface waters was also described by Tsai et al.^56^.

The evolutionary analysis confirmed that the obtained sequences clustered explicitly with other *A. baumanni* sequences from clinical sources and different geographical regions of the world. Ultimately, the majority of research have centered on the “comparative genomics of clinical strains, but it is not known whether the diversity found among clinical *A. baumanni*i strains is representative of the whole *A. baumanni*i population diversity, particularly since *A. baumanni*i can be isolated, although with low (< 10%) recovery rates, from the soil, water, vegetable, and animal sources^21^. We conclude that this approach has made available some important techniques for the exploration of the evolution of *A. baumanni*i as a worldwide infectious agent.

## Conclusion

About 79% of class 1 integrons was found in MDR *A. baumanni* strains from surface water samples in the “Eastern Cape Province, South Africa”. We report the gene cassette *dfrA17, aadA2, aadA5, aadA12, aac (6')-Ib',* and *ant (2'')-I* first appeared in *A. baumannii* strains from environmental samples. The detection of integrons of class 1 and class 2 is related to clinically important mobile genetic elements that are common in clinical settings. Control and monitoring of antimicrobial resistance, with the inclusion of integron appraisal as an indicator of resistance acquisition, can be a major strategy against antibacterial resistance. A more detailed evaluation of the phylogenetic variation of non-clinical *A. baumanni*i strains should contribute better grasp of the steps leading up to the relatively new development of this species as a world's infectious agent.

## Supplementary Information

Supplementary Information.

## Data Availability

The datasets analyzed in this study are available in GenBank with accession number ON 622475-ON622478.
