# Supplementary material for: Prevalence of classes 1 and 2 integrons in multidrug-resistant Acinetobacter baumanni isolates recovered from some aquatic environment in South Africa
Source: Sci Rep. 2022 Nov 24;12:20319. doi: 10.1038/s41598-022-24724-2 (PMC9700688; doi:10.1038/s41598-022-24724-2)
Supplement: Supplementary file 1 — Supplementary Information. [file 41598_2022_24724_MOESM1_ESM.docx]

**Table S1: Drug combination results of *Acinetobacter baumanni***

| **Organism** | **Drugs in combination** | **Single MIC (µg/ml)** | **Combination MIC (µg/ml)** | **FIC (µg/ml)** | **Types of Interaction** |
| --- | --- | --- | --- | --- | --- |
| *Acinetobacter baumanni* (30) | Drug A CIP | 128 | 64 | 1 | Additive |
|  | Drug B COL | 16 | 8 |  |  |
| Acinetobacter baumanni (44) | Drug A CIP | 128 | 8 | 0.31 | Synergistic |
|  | Drug B COL | 32 | 8 |  |  |
| *Acinetobacter baumanni* (30) | Drug A CIP | 128 | 64 | 0.75 | Additive |
|  | drug B CAZ | 256 | 64 |  |  |
| Acinetobacter baumanni (44) | Drug A CIP | 128 | 64 | 0.52 | Additive |
|  | drug B CAZ | 512 | 8 |  |  |
| *Acinetobacter baumanni* (30) | Drug A CAZ | 256 | 128 | 1 | Additive |
|  | drug B COL | 16 | 8 |  |  |
| Acinetobacter baumanni (44) | Drug A CAZ | 512 | 8 |  |  |
|  | drug B COL | 32 | 16 | 0.52 | Additive |
| *Acinetobacter baumanni* (30) | Drug A TET | 8 | 4 |  |  |
|  | drug B COL | 16 | 1 | 0.56 | Additive |
| Acinetobacter baumanni (44) | Drug A TET | 2048 | 1024 |  |  |
|  | drug B COL | 32 | 8 | 0.75 | Additive |
| *Acinetobacter baumanni* (30) | Drug A MEM | 1 | 0.5 |  |  |
|  | drug B COL | 16 | 8 | 1 | Additive |
| Acinetobacter baumanni (44) | Drug A MEM | 16 | 16 |  |  |
|  | drug B COL | 32 | 0.5 | 0.53 | Additive |
| *Acinetobacter baumanni* (30) | Drug A MEM | 1 | 0.5 |  |  |
|  | drug B TET | 8 | 4 | 1 | Additive |
| Acinetobacter baumanni (44) | Drug A MEM | 16 | 8 |  |  |
|  | drug B TET | 2048 | 512 | 0.75 | Additive |
| *Acinetobacter baumanni* (30) | Drug A MEM | 1 | 0.5 |  |  |
|  | drug B CAZ | 128 | 64 | 1 | Additive |
| *Acinetobacter baumanni* (30) | Drug A MEM | 1 | 0.5 |  |  |
|  | drug B CIP | 128 | 64 | 1 | Additive |
| Acinetobacter baumanni (44) | Drug A MEM | 16 | 0.5 |  |  |
|  | drug B CIP | 128 | 64 | 0.53 | Additive |


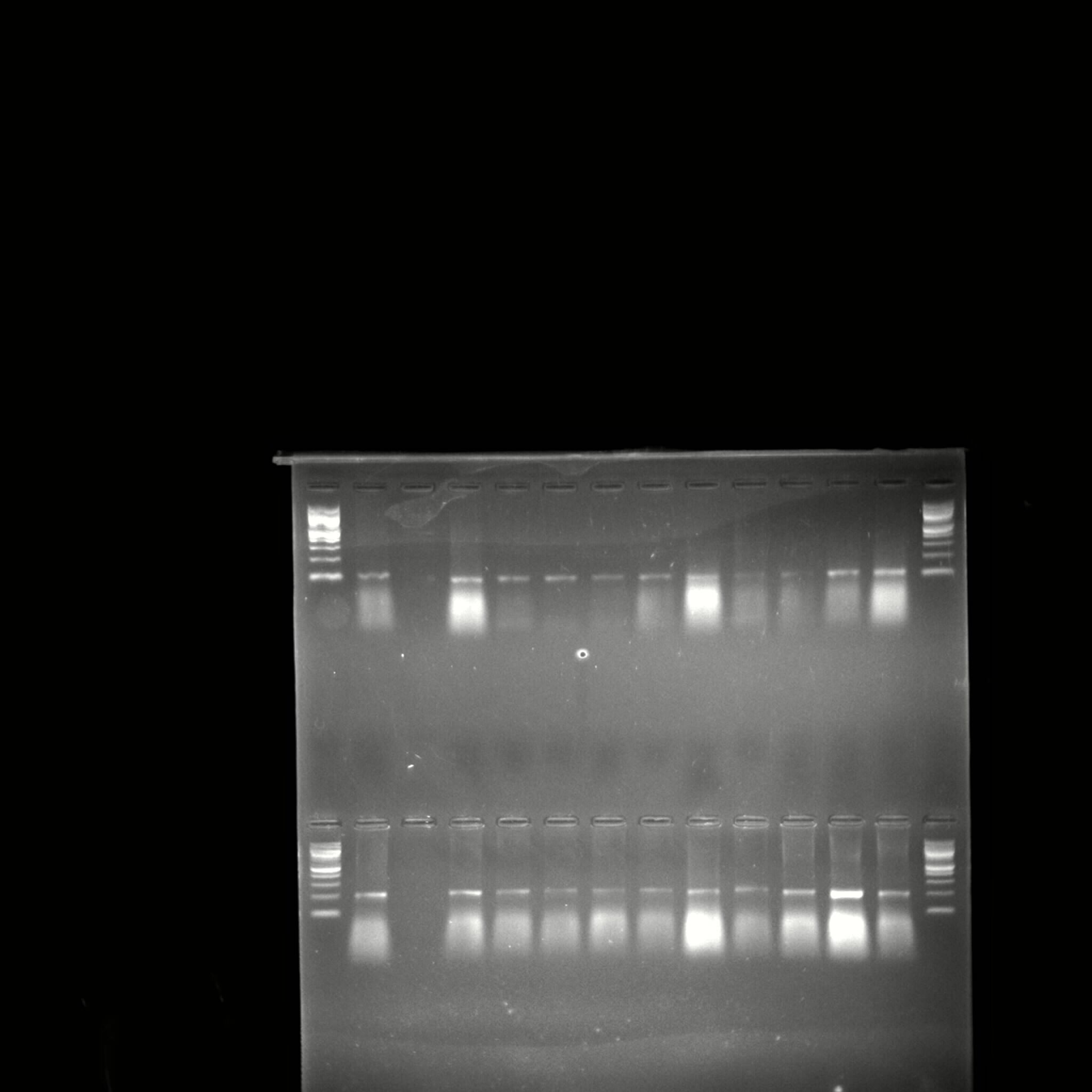


**100bp**

**208bp**

**M L N 1 2 3 4 5 6 7 8 9 10 M**

**Figure S1: The gel electrophoresis picture identified *gyrB* gene of *Acinetobacter baumanni* at 208bp**

M- DNA LADDER (100BP), L- POSITIVE CONTROL –ATCC 19606 N- Negative Control , LANE 1-10 positive isolates


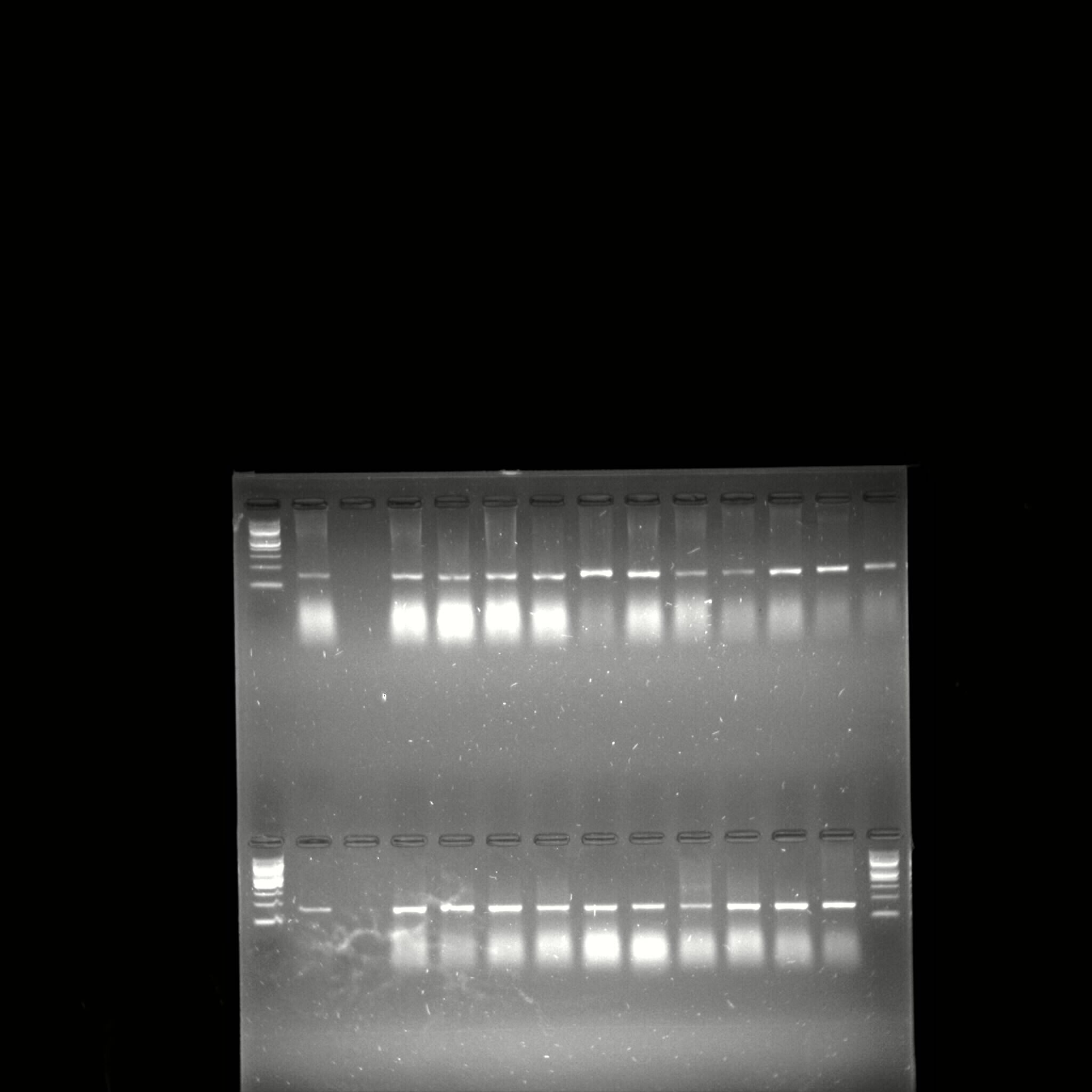


**M L N 1 2 3 4 5 6 7 8 9 M**

**100bp**

**164bp**

**Figure S2: The gel electrophoresis picture of class 1 integron at 164bp**

M- DNA LADDER (100bp), L- POSITIVE CONTROL –ATCC 19606- N- NEGATIVE CONTROL LANE 1-9 POSITIVE ISOLATES


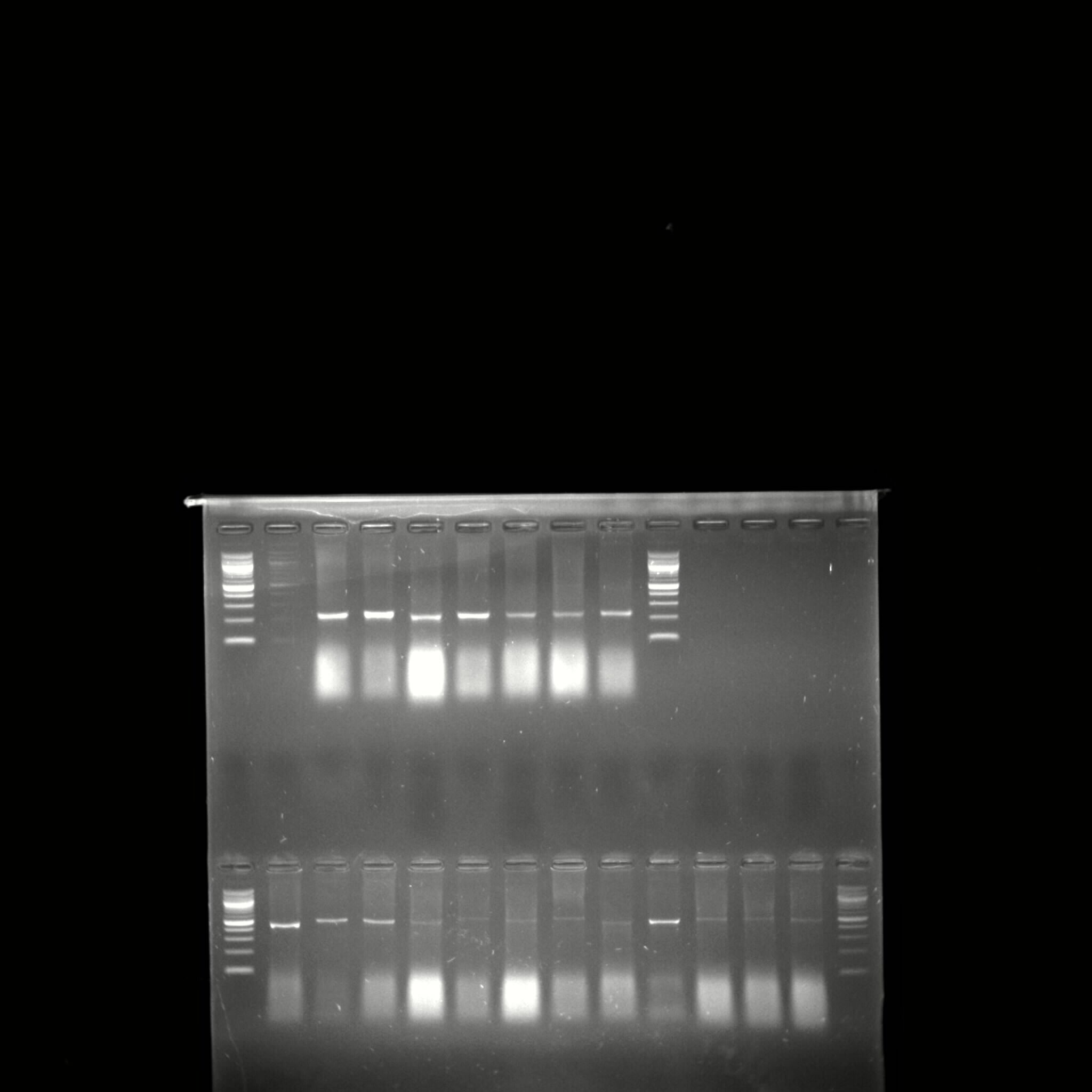


**M N 1 2 3 4 5 6 7 M**

**232bp**

**100bp**

**Figure S3: The gel electrophoresis picture of class 2 integron at 232bp**

**M- DNA LADDER (100BP), N- NEGATIVE CONTROL LANE 1-7 POSITIVE ISOLATES**

**FULL GEL LENGTH**


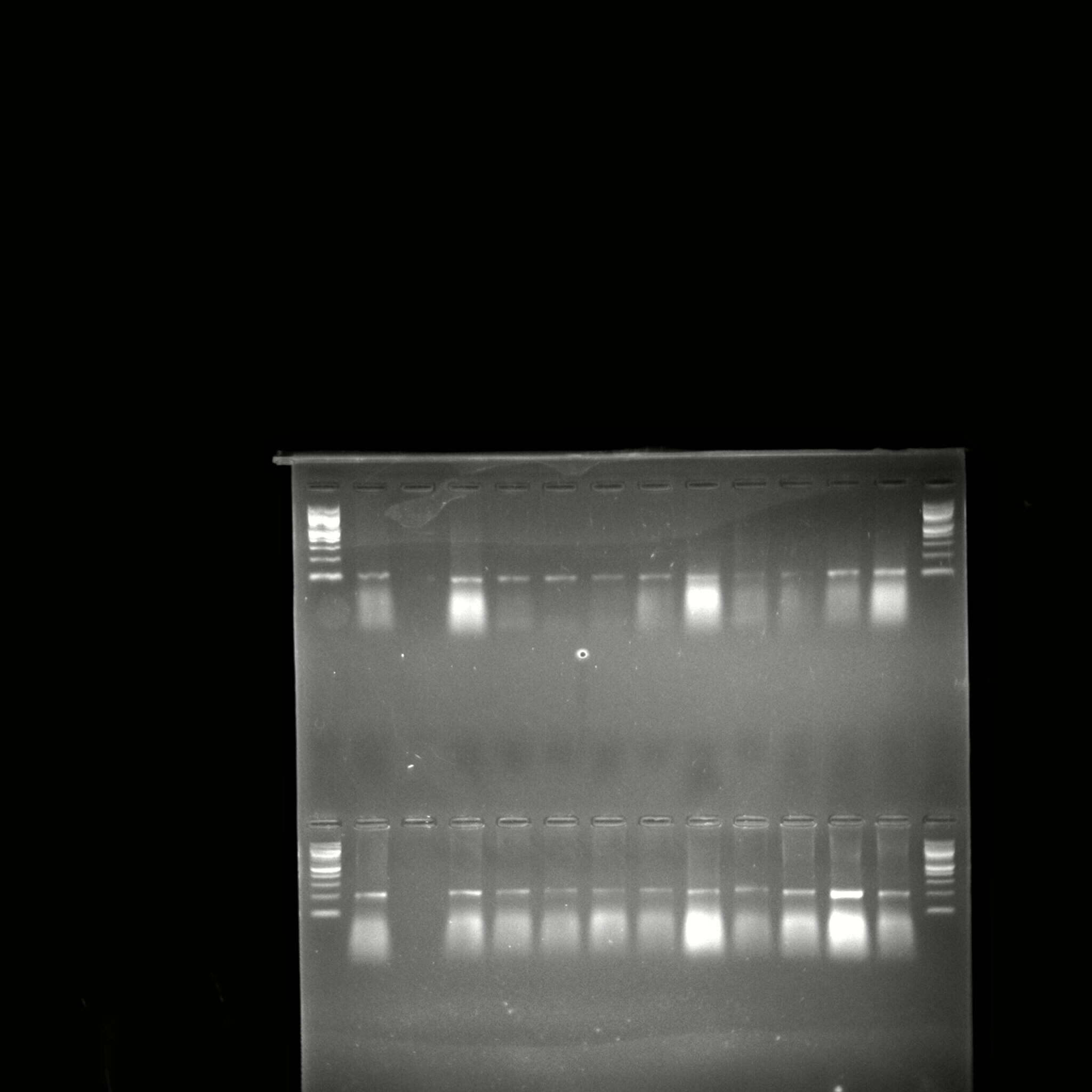


**Figure S4: *Acinetobacter baumanni* gel picture (the arrow shows gyrB gene for A. baumanni at 208bp)**


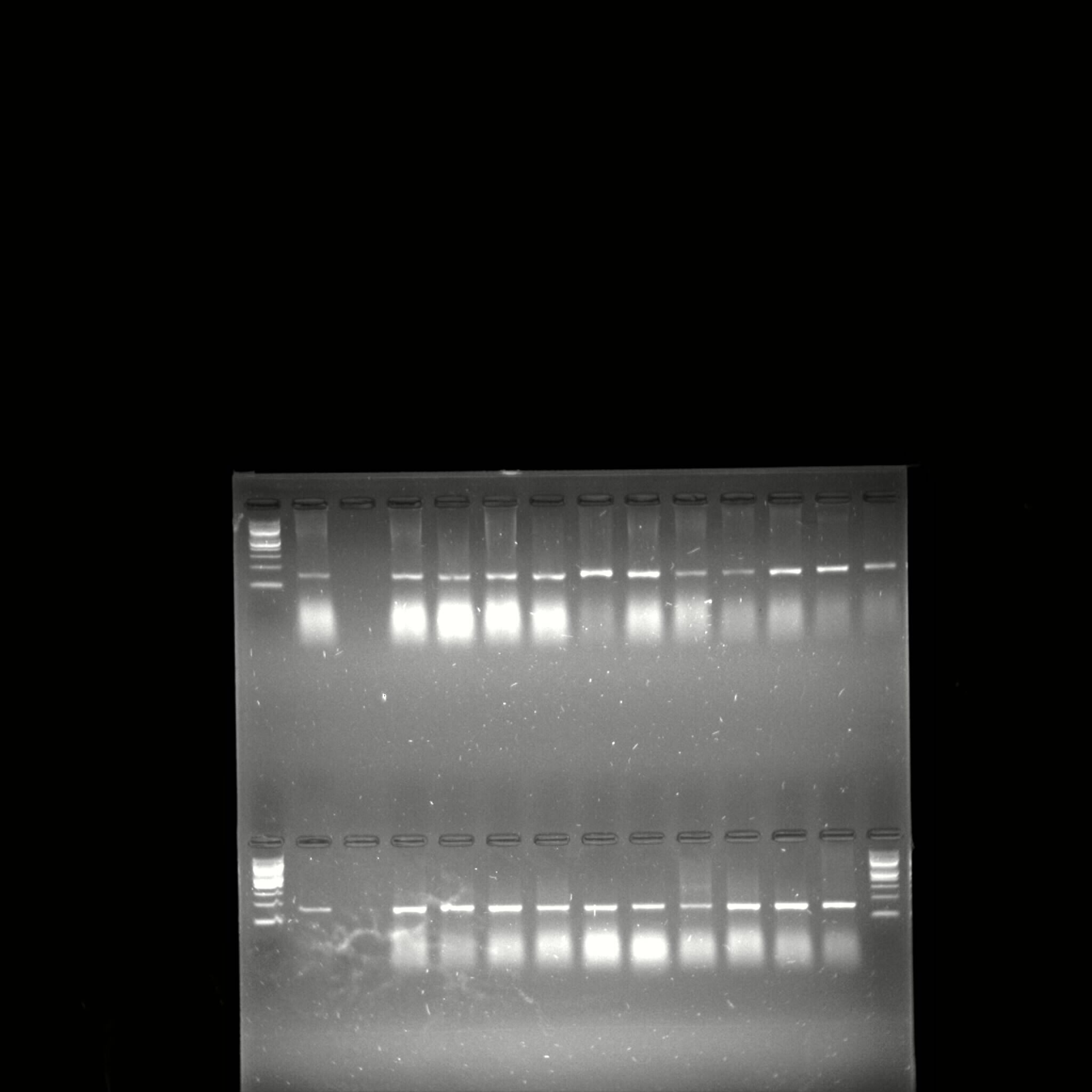


**Figure S5: Class 1 integron at 164bp**


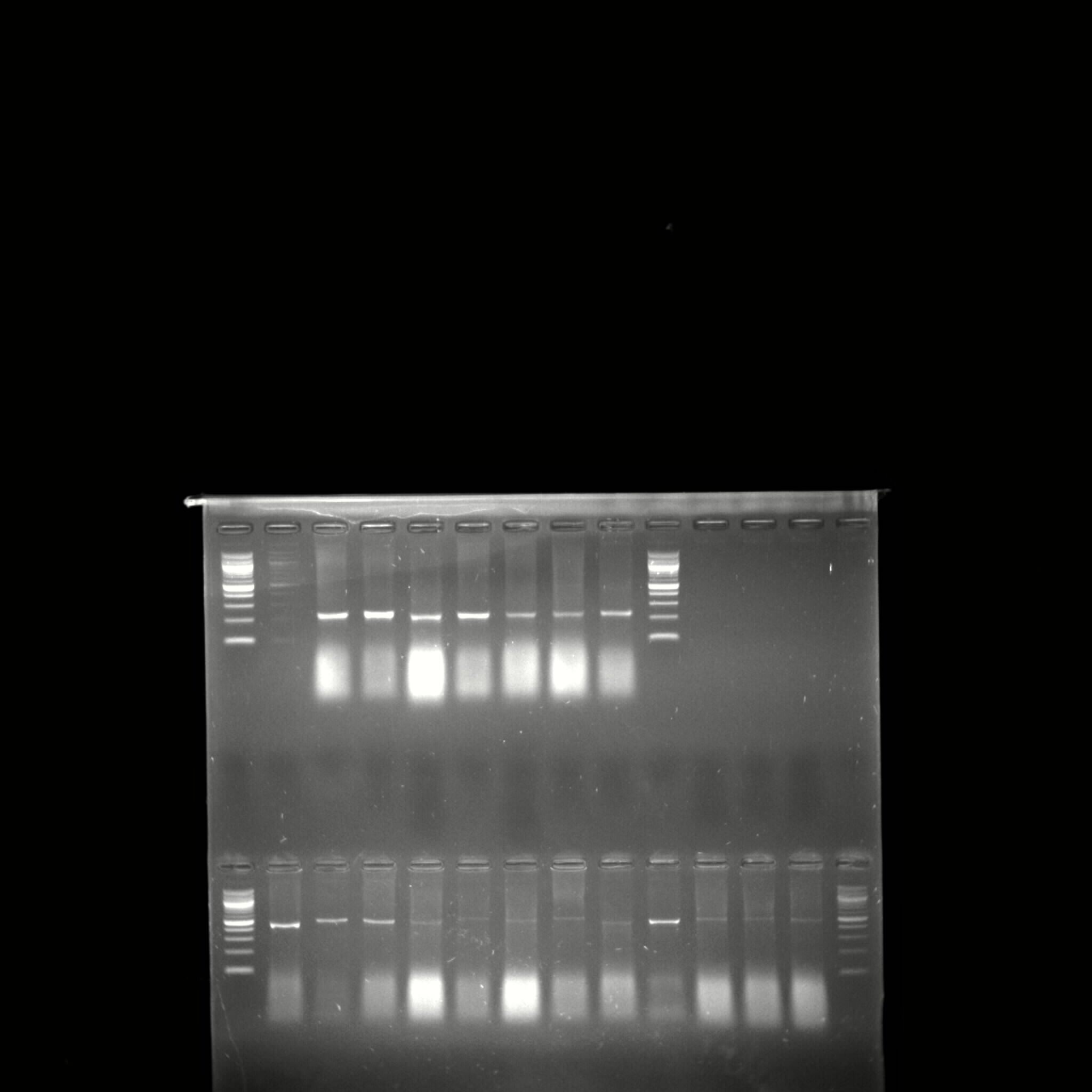


**Figure S6: Class 2 integron at 232 bp (the arrow denotes class 2 integron)**

​
